# Microneedles for Melanoma Therapy: Exploring Opportunities and Challenges

**DOI:** 10.3390/pharmaceutics17050579

**Published:** 2025-04-28

**Authors:** Lufuno Nemakhavhani, Heidi Abrahamse, Sathish Sundar Dhilip Kumar

**Affiliations:** Laser Research Centre, University of Johannesburg, Johannesburg 2028, South Africa

**Keywords:** melanoma, microneedles, skin cancer, 3D printing, biocompatible, cancer

## Abstract

Melanoma is a type of skin cancer that originates in the melanocytes, the epidermis’ basal layer. The skin has traditionally been an attractive administration location for drug delivery in tumor therapy, and it is composed of three layers: the outermost stratum corneum (SC), the middle epidermis, and the deepest layer, the dermis. Melanoma can be treated using a variety of methods, such as chemotherapy, surgery, radiotherapy, and biological therapy, but all are expensive and have side effects. Furthermore, the SC is the primary barrier that contributes to the impermeability of the skin, which is a limitation in epidermal drug transport and can aid in achieving effective drug concentration with minimal side effects at the target location. Microneedles (MNs) are tiny needles that are easy to use, inexpensive, and non-toxic. In recent years, MNs have been significantly studied for the treatment of melanoma due to their excellent biocompatibility, minimal invasion, high patient compliance, simple penetration process, and high SC penetration rate. Most notably, MNs can provide efficient and seldom unpleasant delivery carriers and synergistic effectiveness by combining multi-model techniques with immunotherapy, gene therapy, photodynamic therapy (PDT), and photothermal treatment (PTT). This review will focus on biocompatibility, biodegradability, limitations, fabrication materials, release mechanisms, and delivery of the therapeutics of MNs for melanoma treatment.

## 1. Introduction

Melanoma is an aggressive and invasive skin cancer renowned for its inferior patient survival rate and proclivity to return [1]. Melanoma is a cancer that is becoming more prevalent in the Western world because eumelanin, which is present in higher concentrations in dark-tanned individuals, offers greater UV protection than pheomelanin [2]. The Global Cancer Observatory (GLOBOCAN) estimates that there were 331,647 cases of melanoma worldwide in 2022 and 58,645 deaths [3]. Despite the availability of various treatments for melanoma, including chemotherapy, radiation therapy, immunotherapy, and surgical excision, resistance to current treatments remains a significant challenge, especially when the tumor has metastasized [2]. Furthermore, the above-mentioned treatments often cause undesirable side effects on normal tissues. Therefore, it is essential to develop new treatments that are both more effective and less harmful to patients [4]. There are several types of drug delivery routes commonly used, such as oral drug delivery, hypodermic injections, and transdermal drug delivery (TDD) [5]. Physicians frequently employ oral drug delivery. However, it is not always feasible due to inadequate drug absorption via the gastrointestinal system [6]. The primary drawbacks of administering hypodermic injections include the risk of infection, discomfort during administration, patient dread, anxiety, and incompetence [7]. The TDD system allows drugs to be absorbed through the skin [8]. The skin offers key advantages such as a drug delivery route, including a large surface area and bypassing first-pass metabolism, which improves drug bioavailability and reduces side effects [9].

Transdermal drug delivery (TDD) is achieved through the use of transdermal or topical patches. However, TDD with these products is restricted because of their physicochemical qualities, which inhibit their penetration through the lipophilic stratum corneum (SC) and limit the diffusion rate through the skin. To overcome these issues, microneedles (MNs) have been created to enter the SC in a less invasive way, forming micro-conduits that allow for more efficient drug delivery from the skin surface into the underlying tissue [10]. MNs are a TDD system that combines the technology of transdermal patches with hypodermic needles. The needles are hundreds of microns long, causing little to no discomfort [7]. MNs have been extensively studied by many researchers for drug delivery via the transdermal route and for overcoming the limitations of conventional treatment [11]. This article focuses on microneedles for melanoma treatment, highlighting opportunities and challenges. This review article is organized into five sections. Section 1 introduces the study, while Section 2 explores melanoma and current treatment strategies. Section 3 delves into the background, limitations, mechanisms, types, and fabrication materials of microneedles. Section 4 focuses on their application in melanoma treatment. Section 5 discusses the biodegradability and biocompatibility of microneedle materials, with future perspectives and conclusions.

## 2. Melanoma and Current Treatments

Melanoma is the most aggressive and fatal form of skin cancer [12]. It is a type of cancer that originates in melanocytes, which are the cells responsible for producing melanin in the basal layer of the epidermis [13]. Melanocytes derived from migratory embryonic cells are called neural crest cells and undergo malignant transformation, leading to the production of numerous factors and signaling molecules that promote metastasis and migration. The primary cause of melanoma is exposure to ultraviolet (UV) radiation. UV exposure leads to the formation of deoxyribonucleic acid (DNA) photoproducts, most notably thymidine dimers, which, if not repaired by nucleotide excision repair, result in mutations in cell signaling molecules and DNA replication, ultimately leading to carcinogenesis [14]. Metastatic melanoma refers to cancer that has spread to other parts of the body, such as the bones, lymph nodes, brain, and lungs, making this type of cancer particularly lethal and difficult to treat [1]. Therefore, a thorough understanding of this type of skin cancer is crucial for the successful treatment of metastatic melanoma.

### 2.1. Types of Melanomas

The melanocyte is the malignant cell involved in melanoma. Melanoma typically appears on the skin due to melanocytes located in the epidermis’ basal layer. While melanomas are often pigmented, they can sometimes be presented as red or pink lesions. They may resemble malignant or benign skin lesions [15]. Malignant melanoma is divided into four types: acral lentiginous melanoma, nodular malignant melanoma, superficial spreading melanoma (SSM), and lentigo malignant melanoma [16].

The most common type of melanoma is SSM, accounting for 60–70% of all melanomas, particularly among those with skin phototypes I and II. These phototypes tend to burn easily and either do not tan or tan very minimally [17].

SSM usually appears on the lower extremities in women and on the trunk in men [15]. SSM often manifests as a flat, slowly developing, irregular lesion with variegated pigmentation that enlarges in a radial pattern unless dermal invasion occurs, which is generally indicated by the presence of an elevated region [18]. Nodular melanomas are commonly found on the head and neck of the elderly; they develop rapidly and are often hard, symmetrical, and uniformly pigmented papules or nodules that can ulcerate and bleed [19]. Acral lentiginous melanomas typically appear on the soles, palms, and beneath nails, and are considered unrelated to sun exposure. Although rare among Caucasians, they are more frequent in those with Asian or pigmented skin. These melanomas are usually present as large, pigmented macules, although they can also resemble warts with a verrucous, non-pigmented appearance [20,21]. Lentigo maligna develops as a slow-growing pigmented macule that can remain in situ for many years. When it becomes invasive, it is known as lentigo malignant melanoma and can grow quickly, frequently being poorly defined and variably pigmented; they are more common in adults over the age of 60 [19].

### 2.2. Current Treatments Used for Melanoma

Melanoma is notorious for its high recurrence rate, poor prognosis, and multidrug resistance [22]. Melanoma resistance to therapies is partly attributable to tumor heterogeneity, which enhances their aggressiveness and survival [23]. Surgery, chemotherapy, radiation, and immunotherapy are all potential treatment options for melanoma, depending on its stage, genomics, and location; however, these treatments often come with significant side effects [12]. These side effects include nausea, neuropathy, fatigue, alopecia, vomiting, gastrointestinal toxicity, and myelosuppression [1,24,25]. Metastatic tumors can be surgically removed in certain cases; however, surgical therapy in the setting of established melanoma illness is not curative and does not preclude alternative methods [26]. In chemotherapy, alkylating agents can also induce conditions such as hemorrhagic cystitis, which produces microscopic hematuria, nocturia, dysuria, and mild to severe suprapubic pain [27]. In radiation therapy, there is a large amount of exposure that causes radiation dermatitis [28]. CAR-T cell therapy is a type of immunotherapy that has limited efficacy against solid tumors. This limitation is mostly due to the immunosuppressive tumor microenvironment (TME), which induces a lack of nutrition, hypoxia, necrosis, T-cell fatigue, and other immunosuppressive chemicals including TGF-β [29,30]. Furthermore, these treatments have drawbacks such as off-target toxicity and low bioavailability, which can result in the destruction of neighboring healthy cells [31]. One of the biggest obstacles in melanoma treatment is limited drug penetration, which results in the partial treatment of cancer cells due to the unique properties of melanoma related to tissue architecture and blood vessel structure [32]. Additionally, there is a lack of genetic alterations that can be targeted by currently accessible treatments, which limits the options for effective melanoma therapy [33].

## 3. Microneedles

Microneedles (MNs) are microscale arrays that measure 150–1500 μm in height, 50–250 μm in breadth, and 1–25 μm in tip thickness [34]. They are designed to penetrate the skin’s SC layer without stimulating nerves or causing damage to blood vessels [35]. Consequently, MNs offer multiple advantages when employed in TDD, such as effective drug delivery, ease, improved drug bioavailability, and low invasiveness [36]. MNs are classified into five types: coated, dissolving, solid, hydrogel-forming, and hollow [37]. The classification of MNs is based on their fabrication material, structure, overall form, array density, size, tip shape, and application [38]. MNs are used to transport a variety of chemicals with different molecular weights, such as DNA, chemotherapeutic drugs, vaccines, proteins, polypeptides, and ribonucleic acid (RNA) [39].

### 3.1. Background of Microneedles

Gerstel and Place developed the first MN technique for drug delivery in 1971. During that period, they referred to MNs as “puncturing projections”. The first successful attempts at using MNs came in the 1990s [40]. In the late 1990s, MNs became the focus of substantial research due to advances in microfabrication technology that enabled their production [41]. In 1998, a revolutionary strategy for TDD was developed that significantly improved molecule mobility across the skin [42]. MNs microfabricated by etching micron-sized needle arrays onto silicon increased calcein permeability by more than 1000-fold when inserted into the skin [43]. These MNs offer various compelling benefits, including ease of use, ease of fabrication in accordance with compendial standards, and reduced irritation and pain without severe skin rapture. Additionally, they promote a quicker healing rate than conventional techniques, bypassing the drug’s first-pass metabolism, and achieve the requisite pharmacodynamics and pharmacokinetics responses [44]. This suggested that the use of MNs could be significantly enhanced, which piqued researchers’ interest regarding this treatment modality [43].

### 3.2. Limitations of MNs

MNs have shown promise as a potential therapeutic modality for melanoma but there are several drawbacks such as lower dosage accuracy, the need for careful use of the device to prevent particles from bouncing off the skin surface, and the variation in the thickness of the SC and other skin layers among individuals, which may affect particle penetration depth. Delivery can also be influenced by extrinsic factors such as skin hydration. Repetitive injections may lead to vein collapse, the tip of the MN might break off and stay beneath the skin after the patch is removed, only a tiny quantity of a drug (less than 1 mg) can be administered by bolus, and compressed dermal tissue may obstruct hollow MNs [45].

### 3.3. Mechanism of Microneedles

**Figure 1:**
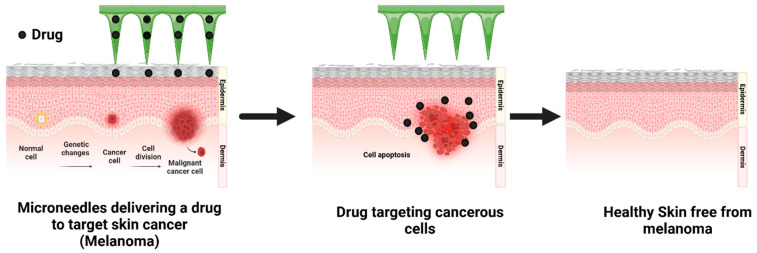
Mechanism of drug delivery using microneedles. Created in BioRender. Dhilip kumar, S. (2025); https://BioRender.com/r23e912.

A typical mechanism of drug delivery using MNs is illustrated in Figure 1. An MN device is constructed by arranging hundreds of MNs in arrays on a small patch to deliver a sufficient amount of drug to provide the desired therapeutic response. The MNs penetrate the SC, bypassing the barrier layer [11]. The drug is directly deposited in the epidermis layer, which subsequently enters the system’s circulation and demonstrates a therapeutic action when reaching the target site [46,47]. Drug delivery in the skin occurs by diffusion across skin layers, which is enhanced by skin appendages. TDD recognizes a variety of permeation pathways, including intracellular, transcellular, and transappendageal. In the intracellular route, most uncharged lipophilic drugs pass via the lipid matrix found between the SC cells. In the transcellular route, the drug moves through hydrated keratinized corneocytes, forming a hydrophilic channel. Drugs are partitioned and distributed throughout keratinized corneocytes, lipid matrices, and cell cytoplasm [48]. The transappendageal pathway involves drug delivery through sweat glands, oil glands, and hair follicles. However, although these accessories cover 0.1% of the body’s surface, they nonetheless provide a direct channel for drugs to enter general blood circulation [48].

### 3.4. Types of Microneedles

**Figure 2:**
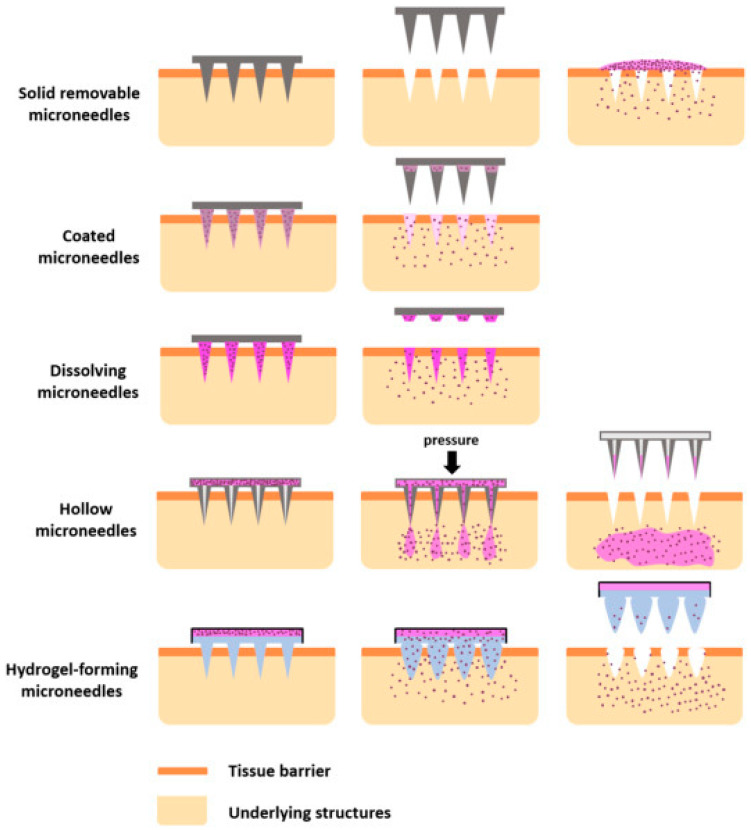
Different types of microneedles; reproduced with permission [35], copyright 2018, Elsevier.

Solid MNs are commonly used to pretreat the skin by generating micron-sized pores. These pores are created when the needles puncture the skin, forming channels through which the drug can directly penetrate the skin layers when a drug patch is applied, thereby boosting penetration [11]. Once applied, the drug is absorbed by capillaries, leading to a systemic effect that can also produce a localized response [49]. Drug delivery via solid MNs occurs through passive diffusion [50]. Solid silicon MNs were fabricated using the tetramethylammonium hydroxide etching process. The resulting MNs had a base width of 110.5 μm and an average height of 158 μm [51]. Li et al. studied polylactic acid MNs and discovered that these biodegradable polymer MNs have sufficient mechanical strength to penetrate the SC and enhance drug adsorption. MNs with a depth of 800 μm and a density of 256 MNs per cm^2^ were found to improve drug permeation [49]. There are different types of MNs illustrated in Figure 2.

Hollow MNs are sub-millimeter devices that penetrate the SC to deliver drugs into the epidermis or dermis [52]. Hollow MNs can transfer drugs through their lumen and release them quantitatively using an external pump [53]. If a rapid bolus injection is required, the flow rate and pressure can be adjusted. These MNs may accommodate a large dosage of the drugs because more drugs can be stored in the hollow area inside the needle. Maintaining a consistent flow rate is essential [54].

A coated MN array consists of sharp micrometer-sized needle shafts that are attached to a base substrate and have their surfaces coated with a drug [55]. In coated MNs, the drug dissolves from the coating layer and is rapidly administered. The amount of drug that can be loaded depends on the needle size, which is typically small, and the thickness of the coating layer [49]. In one study, individually coated MN patches co-delivered various agents with different physicochemical characteristics (immiscible, proteins, molecules, and nanoparticles) in distinct spatial patterns within the skin. MN loading varied by changing the number of coating layers, and different agents were co-delivered into porcine skin. The uniquely coated MNs allow for the co-delivery of multiple distinct compounds and formulations in the skin, with needle-by-needle spatial control [56].

Dissolving MNs are usually fabricated by encapsulating the drug within biodegradable polymers [57,58]. Upon penetration of the SC, the polymer comprising the needle architecture degrades and releases the drug contained within. Dissolving MNs are applied in a one-step process, as the MN remains in place after application [59]. Effective drug delivery is a crucial consideration in the creation of dissolving MNs. Therefore, the mixing of the polymer and drug is a key step in fabrication [50]. Dissolving MNs requires time to dissolve, and achieving full insertion can be challenging. Zhu et al. developed dissolving MNs with encapsulated drugs mounted on solid poly (lactic acid) (PLA) MNs. These MNs demonstrated over 90% drug delivery efficiency within 30 s, compared to typical MNs, which took 2 min. In vivo tests revealed that microholes created by the dissolving process were entirely formed within 1 h, indicating that these MNs are safe to use [60]. Hydrogel-forming microneedles (HFMs) are composed of swellable polymers (crosslinked hydrogels) and function differently from other types of MNs. Due to the hydrophilic nature of the material, HFMs expand upon application to the skin, allowing them to readily absorb water. This property makes them valuable in biological applications such as interstitial fluid absorption, which occurs in the tissue spaces surrounding cells, particularly in the dermis layer of the skin [61]. HFMs are considered minimally invasive as they are on the microscale and do not penetrate deeply enough into the skin to interact with and stimulate pain receptors located deeper into the dermis [62].

### 3.5. Fabrication Materials and Methods of Microneedles

**Figure 3:**
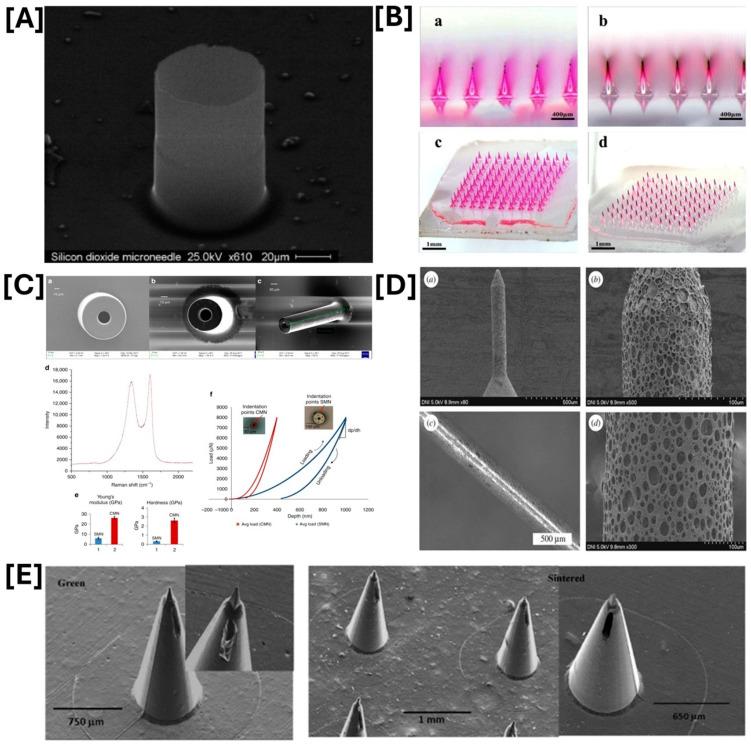
Types of fabrication materials of microneedles: (**A**) silicon dioxide microneedles—reprinted from reference [66], copyright 2011, Elsevier; (**B**) polymer (hyaluronic acid (**a**) side view and (**c**) top view and polyvinylalcohol (PVA) (**b**) side view and (**d**) top view)—reprinted from reference [67], copyright 2020, Elsevier; (**C**) SU-8 microneedle (SMN), pyrolyzed MN and glassy carbon microneedle ((**a**) Scanning electron micrograph of an SMN (outer diameter 100 μm, inner diameter 50 μm). (**b**) Corresponding pyrolyzed MN. (**c**) Tilted view of the same CMN. (**d**) Raman spectrum of the carbon microneedle. (**e**) Comparison of Young’s modulus and hardness for the SU-8 and carbon MNs. (**f**) Load vs. displacement data for an SMN and corresponding pyrolyzed CMN)—reprinted from reference [68], copyright 2018, Elsevier; (**D**) porous polymer coatings on metal microneedles (MN) both type A and type B, (**a**,**b**)—SEM micrograps of a type a MN, (**c**) Stereo image of a type B MN, and (**d**) SEM images of a type B MN—reprinted from reference [69], copyright 2018, Royal Society; and (**E**) sintered alumina microneedles—reprinted from reference [70], copyright 2016, Elsevier.

The primary function of MNs is to penetrate the skin or other biological tissues without breaking or bending. An ideal MN design should exhibit a low insertion force and a high fracture strength. Several factors must be considered when designing MNs for skin penetration: geometric properties (shape, tip, size, length, and diameter), physical form (beveled tip, hollow, conical, side-opened, and solid), material, application, fabrication feasibility, array architecture, the total number of MNs, and surface layer properties (hydrophobicity) [63]. To achieve these characteristics, several parameters may be optimized, such as the choice of fabrication material [41]. Metals, silicon, ceramics, polymers, glass, and polysaccharides are the six common materials utilized to fabricate MN [11]. Recently, polymer materials such as hyaluronic acid, polyvinyl alcohol (PVA), carboxyl methylcellulose (CMC), polylactide-co-glycolide (PLGA) gelatin, and polyvinyl pyrrolidone (PVP) have been used for the fabrication of MNs. Figure 3 illustrates different types of MN materials: (A) silicon dioxide microneedles, (B) polymer (hyaluronic acid and polyvinylalcohol (PVA)), (C) SU-8 microneedle (SMN), pyrolyzed MN and glassy carbon microneedle, (D) porous polymer coatings on metal microneedles, and (E) sintered alumina microneedles. MN designs also vary depending on the application and fabrication method employed [63]. Various fabrication methods have been employed, including machining with chemical etching, photolithography with selective etching, laser cutting, micro-milling, micro-injection, ceramic sintering, two-photon polymerization, multiple-pulse laser microhole drilling, soft lithography, and laser machining [64,65]. Table 1 summarizes the fabrication materials and methods used to create different types of MNs.

## 4. Microneedles in Melanoma Treatment

### 4.1. Transdermal Drug Delivery—Stratum Corneum

The transdermal route is employed in various clinical applications to overcome the significant limitations associated with oral drug delivery techniques [75]. Human skin acts as a protective barrier to the external environment; its highly specialized structure allows for one-way channelization [76]. The skin has three layers: epidermis, dermis, and hypodermis [77]. TDD is the process of introducing drugs into the bloodstream or systemic circulation through various layers of the skin [76]. However, the application of TDD has been limited to potent, small, and moderately lipophilic molecules due to the inert, lipophilic, and compact structure of the SC layer of the skin [78,79]. The SC is the uppermost layer of the epidermis; it consists of 15 or more layers of flattened corneocytes surrounded by a lipid-rich extracellular matrix [80].

Microneedles (MNs) are known to penetrate the epidermis, superficial dermis, and SC, dissolve rapidly in the interstitial fluid of the skin, create microchannels in the skin, release encapsulated drugs within minutes, and can administer a broad range of therapeutic agents into the skin layers for systemic or local delivery [81,82]. Nguyen et al. assessed the MN’s ability to penetrate the SC. Poly (vinyl alcohol) (PVA) MNs were designed, evaluated, and applied to human cadaver skin to improve doxorubicin (Dox) TDD in vitro. In vitro permeation studies revealed that the MN-treated skin exhibited significantly higher drug permeability compared to the untreated group. The location of the drug within the needle array was found to influence both its release profile and its penetration into and through human skin [83].

### 4.2. Nanoparticles Utilized with Microneedles

Nanoparticles (NPs) have been widely used to deliver conventional drugs, nucleotide vaccines, and recombinant proteins [84]. NP formulations exhibit distinct size-dependent physicochemical properties and can be composed of a wide range of compounds, including lipids, carbohydrates, both degradable and non-degradable metals, and organic and inorganic compounds. NPs offer several advantages over traditional drug delivery systems. Nanoparticulate systems provide prolonged drug release while protecting encapsulated components from proteolytic and chemical destruction [85]. During TDD, the SC serves as a significant barrier to NP penetration. The epidermis, a stratified epithelium, is located just above the dermo-epidermal junction. Unlike the SC, the epidermis is hydrophilic, which restricts the penetration of lipophilic agents. The presence of proteolytic enzymes, which may degrade foreign materials, combined with tight connections within the skin layers, hinders the adsorption of NPs into the epidermis [86]. Various types of MNs have been employed to enhance the permeability of nanoparticles [41]. The penetration of poly (lactic-*co*-glycolic acid) (PLGA) NPs of various sizes containing non-encapsulated dyes (fluorescein isothiocyanate and Rhodamine B) across excised porcine skin, which had previously been treated with polymeric MN arrays, was investigated. MN-assisted penetration of metallic NPs into an agarose gel mimicking skin was also studied. Factors such as NP composition, particle size, particle surface charge, and the chemical nature of the encapsulating dye all significantly affected NP permeability. The results showed that penetration depths increase as mesh pore size increases, due to the passage of large agglomerates. These particles appeared to cause damage to the target surface. The applied pressure positively influenced penetration depth, which increased as the pressure increased. Additionally, as expected, the use of MNs enhanced the microparticle penetration depth [87].

### 4.3. Synergistic Techniques Utilized with Microneedles

In the treatment of superficial skin tumors (SSTs) such as melanoma, the combination of photodynamic therapy (PDT), photothermal therapy (PTT), chemotherapy, gene therapy, immunotherapy, and numerous other therapeutic techniques is crucial for effective tumor treatment [88,89]. A self-developed mesoporous nanocarrier was combined with phthalocyanine, a near-infrared dye from the second generation of photosensitizers (PSs). MNs are used to enhance therapeutic efficacy by facilitating deeper penetration of the PS into the skin. The results revealed that the penetration was 27.2% for MN-free pretreatment and 63.1% for MN-assisted therapy [90]. Ahmed et al. conducted an in vitro study that involved co-loaded doxorubicin (DOX) and celecoxib (CEL) liposomes delivered using MNs to target B16 murine melanoma cells to improve single-agent chemotherapy. The anticancer efficacy against melanoma in nude mice was greatly improved by utilizing DOX/CEL co-loaded liposomes compared to single-drug-loaded liposomes [91]. Sun et al. employed the same cell line for the chemo-PTT-synergetic approach. A self-assembled nanomicelle-dissolving microneedle (DMN) patch was used to deliver paclitaxel as the primary chemotherapeutic drug and the PS IR780. In vivo results, compared to intravenous injections, showed that IR780 delivered by PTX/IR780-NMs@DMNs to the tumor site resulted in complete drug accumulation. The anticancer results indicated that PTX/IR780-NMs@DMNs successfully eliminated tumors with an 88% cure rate without causing damage to healthy tissue [92].

## 5. The Biodegradability and Biocompatibility of MN Materials

Microneedles (MNs) cross biological barriers and interact with living tissue. Therefore, biocompatibility is crucial when selecting MN material. The biodegradability and biocompatibility of MNs depend on the type of fabrication material [53]. Biocompatible materials do not induce toxicity or immune reactions when in contact with the body or its fluids. When a substance degrades in tissue, its degradation products should be non-toxic. Accumulation of slowly degrading materials in tissues is undesirable. Materials may exhibit different reactions when in contact with living tissue due to variations in temperature and pH conditions compared to the surrounding environment. It is crucial to assess a material’s compatibility with the conditions to which it will be exposed [74].

### 5.1. Biodegradability of MN Materials

Biodegradable MNs, which are typically composed of biodegradable polymers such as PLGA, chitosan, polylactic acid, and polyglycolic acid, form the matrix and degrade in the skin after application, allowing for sustained drug release for months by selecting the appropriate polymer [93]. PGA is a hydrophilic, highly crystalline polymer with a rapid breakdown rate. PLA, while structurally identical to PGA, has differing physical, mechanical, and chemical characteristics due to the inclusion of a methyl group present on the alpha carbon. Generally, the co-polymer PLGA is preferred over its constituent homopolymers because PLGA provides improved control over degrading qualities by altering the monomer ratio. For instance, the breakdown rates of PLGA may vary depending on the chain composition, crystallinity, and hydrophilic/hydrophobic balance [94].

Biodegradable polymeric MNs were developed to facilitate continuous TDD. Hydrogel swelling in response to contact with interstitial fluid following needle insertion facilitates MN separation within the skin. The hydrogel particles immediately absorbed water, causing the MNs to fracture due to volume expansion differences between the needle–polymer matrix and the hydrogel particles. The enlarged particles caused the MNs to break down completely, leaving the tips embedded in porcine cadaver skin in vitro and nude mouse skin in vivo. The drug delivery properties of the hydrogel polymer could potentially enable rapid and sustained drug distribution [95].

Polysaccharides are biodegradable, and their biological activity can be readily adjusted. Most polysaccharide MNs break down in the skin, releasing the encapsulated payload. If absorbed through the skin, they can be progressively removed by the kidneys. Many polysaccharides used in drug delivery are derived from natural sources; chitin and alginate, extracted from crab shells and algae, respectively, are widely used in drug delivery. Chitosan is formed by the deacetylation of chitin, which is the chemical process of removing acetyl groups from chitin and substituting active amino groups. Its polycationic nature allows it to bind to mammal cells firmly and can be employed in hemostasis and spermicidal techniques. Its cationic nature has made it suitable for use in controlled-release formulations [96]. Chitin is biocompatible, and lysozymes degrade it into non-toxic residues by hydrolyzing the acetylated residues [97]. Luo et al. [98] studied gelatin methacryloyl (GelMA) MNs and their ability to release DOX to induce a therapeutic effect on the melanoma cell line A375. The GelMA MNs released the loaded drug through swelling and enzymatic scaffold disintegration. In contrast to the burst release observed in other MN formulations, the GelMA-based MN patch demonstrated a steady release of the loaded DOX, especially at higher crosslinking degrees (30 s and above). This controlled release addressed concerns about burst release and its associated toxicity [98].

### 5.2. Biocompatibility of MN Materials

Biocompatible polymers are increasingly used in biomedical applications. Biocompatible polymers are both synthetic and natural, and they operate in proximity to living systems or in close contact with live cells [99]. These are used to access, treat, enhance, or replace any tissue, organ, or bodily function. A biocompatible polymer increases biological functions without interfering with regular functioning, causing allergies or side effects [100]. Polyglycolic acid is synthesized from glycolic acid by ring-opening polymerization. PGA is very hydrophilic, which improves capillary forces in porous materials. As a result, PGA is regarded as an excellent material for porous MNs due to its high mechanical strength and great absorption capabilities [101]. Si and Si-based materials are extremely strong mechanically, as well as thermally and chemically stable. However, when the Si MN penetrates the skin, fractures occur quickly, and the fractured needle remains in the body, leading to poor biocompatibility and undesirable responses such as inflammation [53]. Silicon (Si) is a suitable material for micromachining because of its crystalline form, which allows for precise etching to achieve highly exact geometries, and the use of established batch production processes pioneered by the semiconductor industry [102]. To enhance biocompatibility, solid Si MNs were coated with gold (Au). The mechanical strength of the manufactured product was determined using the Vickers hardness test. The MNs were observed to penetrate the skin without causing fractures, due to their increased pressure compared to the skin’s resistance [103]. Gold (Au), platinum (Pt), nickel (Ni), palladium (Pd), and silver (Ag) are among the metals utilized in MN fabrication [74]. Metal MNs undergo corrosion, which is influenced by pH, oxygen, and moisture levels, which can vary significantly both inside and outside the body. A metal implant that performs well in one body state may nevertheless exhibit undesirable corrosion in another due to oxidation and acidic erosion. The corrosion of metals is accelerated in the presence of aqueous ions. In normal conditions, most fluids in the human body consist mainly of Na^+^, Cl^−^, 0.9% saline, and other trace ions, along with a variety of amino acids and soluble proteins. At 37 °C, the pH of these solutions is near neutral, ranging from 7.2 to 7.4. However, pH levels in bodily fluids can decrease under certain physiological conditions such as inflammation. The durability of metal implants may also be influenced by factors such as body ion deposition and blood pressure [104]. Au and Pt coatings have been demonstrated to enhance biocompatibility; however, they are generally expensive [74].

## 6. Future Perspectives and Conclusions

This review article discusses MNs for melanoma treatment, highlighting both opportunities and challenges. The application of MNs in melanoma treatment has demonstrated their importance and efficiency, along with their functional roles in synergetic techniques. MNs utilize the TTD strategy to penetrate the skin and deliver drugs to targeted locations. Research has focused on MN fabrication methods and materials, exploring their potential therapeutic applications for treating melanoma. The application of MNs and their synergetic techniques has demonstrated significant therapeutic effects, as documented in the literature. However, there are several drawbacks to MNs, including lower dosage accuracy, the need for careful use to prevent particles from bouncing off the skin surface, repetitive injections potentially leading to vein collapse, and the risk of MN tips breaking off and remaining beneath the skin after the patch is removed. The rise of 3D bioprinting may address some of these drawbacks in melanoma treatment using MNs. Three-dimensional bioprinting involves precisely mimicking the architecture of natural skin, allowing MNs to be tested steadily without the need for complex in vivo studies. Biomimicry and the complexity of bioprinted skin can enhance healing capabilities and provide a detailed biological function for melanoma treatment with MNs. Nanotechnology and photodynamic therapy are current biomedical applications for melanoma treatment that can be utilized. Nanotechnology is the delivery of nanoparticles that contain a drug/photosensitizer (PS). PDT is a therapy that uses tumor-specific PSs and a specific type of light. When exposed to a certain wavelength of light, the PS creates reactive oxygen species, which may affect cancer cells and cause necrosis and death. This type of treatment modality has been shown to be effective against melanoma.

## Figures and Tables

**Table 1:** Fabrication materials used in microneedling.

Fabrication Material	Fabrication Method	Type of Microneedle
Silicon	Lithography and etching	Solid, hollow, and coated MNs [41]
Metal	Laser cutting, electroplating, and photochemical etching	Hollow and coated MNs [53,63]
Polymer	3D printing	Dissolving MNs [67,71,72]
Ceramics	Micro molding	Solid MNs [11,73]
Glass	Micropipette pulling	Hollow MNs [74]
Polysaccharides	Micro molding	Dissolving MNs [59,74]
